# Omental Flap for Thoracic Aortic Graft Infection

**Published:** 2015-07-24

**Authors:** Andrew A. Marano, Adam M. Feintisch, Mark S. Granick

**Affiliations:** Division of Plastic Surgery, Department of Surgery, Rutgers New Jersey Medical School, Newark

**Keywords:** graft, thoracic, aorta, infection, flap, omentum, sternum

**Figure:**
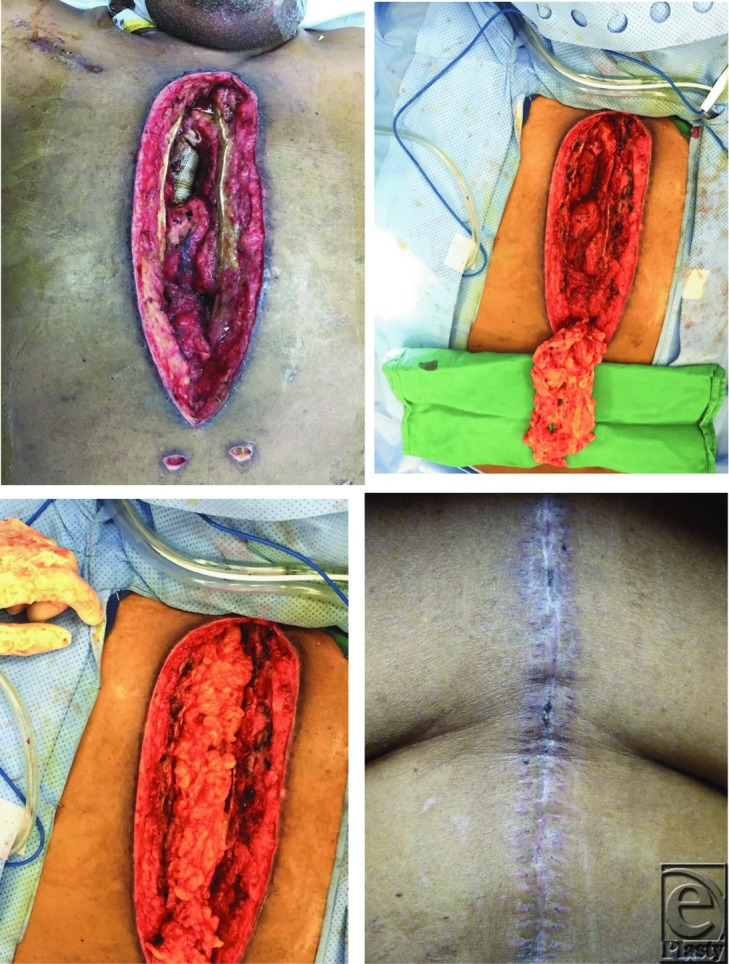


## DESCRIPTION

A 73-year-old man presents 2.5 weeks status postascending aortic and hemiarch synthetic graft replacement with a purulent sternal wound infection.

## QUESTIONS

1. **What is the cause of aortic prosthetic graft infection and how frequently does it occur?**
2. **How do patients with aortic graft infection present and how is the diagnosis made?**
3. **What are the treatment modalities for thoracic aortic graft infection?**
4. **What is the role of the omental flap and what are its advantages over other flaps?**

## DISCUSSION

Prosthetic aortic grafts are frequently used in cardiothoracic surgery to repair aortic aneurysms, dissections, ruptures, or occlusive disease. While only 1% to 3% of these grafts become infected, a mortality rate between 25% and 88% makes early diagnosis and proper management imperative. Although infections may occur via contamination during surgery, transient colonic ischemia, aortoenteric fistulae, and erosions have also been implicated.^1^ *Staphylococcus aureus* and *S epidermidis* are typically responsible for acute and chronic infections, respectively.^2^ The incidence of graft infection may be decreased by avoiding prolonged preoperative hospital stay, administering preoperative prophylactic antibiotics, and careful handling of the graft prior to insertion.^1^

Aortic graft infections present with vague symptoms. Early infections, defined as those occurring within 4 months of surgery, may present with recurrent fevers, leukocytosis, new-onset back or groin pain, and local swelling. Late infections, those more than 4 months after surgery, may lack systemic symptoms and present instead with infection-related complications, such as false aneurysm, graft erosion, and osteomyelitis.^2^ The nonspecific nature of presentation makes imaging studies a necessary adjunct in diagnosing graft infection. Computed tomography allows visualization of tissue planes, perigraft tissue changes, and fluid collections, and is thus the diagnostic test of choice. It is not without shortcomings, however, as it may be difficult to discern changes caused by infection from those due to surgery. Ultimately, aspiration of a perigraft fluid collection or culture of an open wound is needed to confirm the diagnosis and guide management.^1^

The gold standard for surgical management of infected vascular grafts is complete graft explantation, wide debridement of devitalized tissue, and revascularization via extra-anatomic bypass grafting.^3^ In thoracic aortic graft infections, however, anatomic limitations may preclude the use of this surgical modality. Management is, therefore, limited to either in situ graft replacement or graft-preserving techniques. Graft replacement is achieved by complete graft explantation followed by insertion of either a synthetic graft or a homograft. If the infection is limited to only a portion of the graft, partial excision and graft repair may be adequate.^4^ Partial or complete graft replacement, particularly with the use of a synthetic, has had reserved success. A 1999 study detailing outcomes of thoracic aortic graft infection repair reported a 42% in-hospital mortality rate.^5^ Alternatively, graft explantation may be avoided when the infection is caused by low-virulence organisms and is not complicated by false aneurysm or anastomotic leak. Mathes et al^6^ reported success using serial debridement, in situ irrigation with an antimicrobial solution, and locoregional tissue transposition.

Contamination of nearby tissues and the presence of a surrounding dead space may occur following repair of an infected aortic prosthesis regardless of whether the graft is preserved or replaced. Transposition of healthy, well-vascularized tissues can be used to fill this space and prevent reinfection of the graft. Viable options for tissue transposition include omental, rectus abdominis, and pectoral flaps. Omental flaps are particularly suitable for soft tissue reconstruction of this area as they carry a robust vascular and lymphatic supply and cause minimal donor site deformity. They may be dissected using a transabdominal or transdiaphragmatic approach. Although one may encounter limited exposure via a transdiaphragmatic approach, it is associated with a 50% lower rate of ventral hernia when compared to using a midline laparotomy.^7^ Rectus abdominis and pectoralis flaps are also considerations; however, such flaps require muscle sacrifice and carry a significant risk of abdominal or chest wall instability, particularly in patients with sternal nonunions. In a 2013 study, Shah et al^8^ documented the use of omental flaps in 11 patients who underwent either graft-preserving surgery or explantation with homograft replacement. They reported a mortality rate of 0.09%, well below that of the literature, and no complications related to omental flap dissection.^8^ These results support soft tissue coverage with an omental flap as an effective strategy for dead-space elimination and prevention of graft reinfection following repair of thoracic aortic graft infections.

Our patient underwent serial debridements and washouts with antibiotic containing irrigation followed by laparoscopic omental flap dissection and transposition through the inferior most aspect of the sternal wound. The omental flap was wrapped around the ascending aortic arch synthetic graft and bilateral pectoralis major muscle flaps were elevated and transposed toward the midline. The skin was subsequently approximated to allow for complete sternal wound closure, which healed cleanly and without complications.
